# Active surveillance cultures for multidrug-resistant Gram-negative organisms in the intensive care unit: is it necessary to conduct them weekly?

**DOI:** 10.3205/dgkh000645

**Published:** 2026-03-20

**Authors:** Lorena Lima, Elaine Alves, Bianca Santos, Adriana Assumpção, Priscila Oliveira, Priscilla Monteiro, Julia Mascarenhas, Clara Rizzo, Sérgio Morgado, Luiz Mascarenhas

**Affiliations:** 1 Hospital Infection Control Committee, São Francisco na Providência de Deus Hospital, Rio de Janeiro, Brazil; 2 Estácio de Sá University, Rio de Janeiro, Brazil; 3 Federal University of the State of Rio de Janeiro, Rio de Janeiro, Brazil; 4 Laboratory of Molecular Genetics of Microorganisms, Oswaldo Cruz Foundation, Rio de Janeiro, Brazil

**Keywords:** contact precautions, ICU, infection, MDR, active surveillance culture

## Abstract

**Aim:**

Multidrug-resistant Gram-negative bacteria (MDR-GNB) pose a major threat to public health due to the limited treatment options and their frequent association with healthcare-associated infections. Active surveillance culture (ASC), a component of infection prevention strategies, remains controversial. This study evaluated the effectiveness of ASC performed weekly versus only upon ICU admission and discharge for detecting MDR-GNB.

**Materials and methods:**

In a prospective study, two monitoring strategies were compared over a period of 18 months in six intensive care units. Phase 1 involved conducting ASC weekly, while Phase 2 entailed conducting ASC exclusively at the time of ICU admission and discharge.

**Results:**

A total of 233 MDR-GNB infections were documented: 130 (11.38/1,000 patient-days) in Phase 1 and 103 (8.47/1,000 patient-days) in Phase 2. This reflects a statistically significant decrease in infection rates in Phase 2 (IRR: 1.34; 95% CI: 1.04–1.74). No significant differences were observed in species-specific infection rates between the two phases.

**Conclusions:**

Reducing ASC frequency from weekly to only ICU admission and discharge did not increase MDR-GNB infection rates. The implementation of comprehensive infection prevention and control measures proved sufficient for the management of bacterial infections.

## Zusammenfassung

**Zielsetzung:**

Multiresistente Gramnegative Bakterien (MDR-GNB) stellen aufgrund der begrenzten Behandlungsmöglichkeiten und ihrer häufigen Ursache Healthcare-assoziierter Infektionen eine Gefahr für die öffentliche Gesundheit dar. Die aktive Überwachungskultur (ASC), ein Bestandteil von Infektionspräventionsstrategien, ist nach wie vor umstritten. In dieser Studie wurde die Wirksamkeit der wöchentlich durchgeführten ASC im Vergleich zur nur bei Aufnahme und Entlassung aus der Intensivstation durchgeführten ASC zur Erkennung von MDR-GNB bewertet.

**Methode:**

In einer prospektiven Studie wurde über einen Zeitraum von 18 Monaten in sechs Intensivstationen zwei Überwachungsstrategien verglichen. In Phase 1 wurde das ASC wöchentlich durchgeführt, in Phase 2 ausschließlich bei der Aufnahme in die Intensivstation und bei der Entlassung.

**Ergebnisse:**

Es wurden 233 MDR-GNB-Infektionen dokumentiert: 130 (11,38/1.000 Patiententage) in Phase 1 und 103 (8,47/1.000 Patiententage) in Phase 2. Das spiegelt einen signifikanten Rückgang der Infektionsraten in Phase 2 wider (IRR: 1,34; 95 % CI: 1,04–1,74). Es wurden keine signifikanten Unterschiede in den artspezifischen Infektionsraten zwischen beiden Phasen beobachtet.

**Schlussfolgerung:**

Die Reduzierung der Häufigkeit von ASC von wöchentlich auf bei Aufnahme und Entlassung aus der Intensivstation führte nicht zu einem Anstieg der Infektionsraten mit MDR-GNB. Die Umsetzung umfassender Maßnahmen zur Infektionsprävention und -kontrolle erwies sich als ausreichend für die Behandlung bakterieller Infektionen.

## Introduction

Multidrug-resistant Gram-negative bacteria (MDR-GNB), including *Acinetobacter (A.) baumannii* (MDR-AB), *Pseudomonas (P.) aeruginosa* (MDR-PA) and carbapenem-resistant Enterobacteriaceae (CRE), represent pathogens that have become a significant global health concern due to their association with healthcare-associated infections [1], [2]. The paucity of effective treatment options engenders considerable challenges in the management of these infections [3]. Infection prevention and control (IPC) interventions have been implemented in many hospitals with a range of recommendations from committees and societies over the years. These interventions include disinfectant surface cleaning, strict control of antibiotic use, contact precautions), hand hygiene, and tracking or active search swabbing. However, the relative effectiveness of IPC is still inconclusive, especially in high-resistance endemic settings [4]. The active surveillance culture (ASC) program, a component of an infection prevention strategy, remains a subject of debate, and its benefits are contingent on factors such as the target population, the level of endemicity, and the integration of multifaceted strategies [5]. In particular, swab screening for the detection of asymptomatic colonization is laborious and has a low cost:benefit ratio. It also commonly produces false negatives, which increases the risk of contamination of other patients [6]. Therefore, we question the effectiveness of frequent ASC. The aim of this study was to evaluate the performance of ASC in an ICU by testing patients either weekly or only upon ICU admission and discharge.

## Materials and methods

This study was conducted at the São Francisco na Providência de Deus Hospital (Rio de Janeiro/Brazil), a tertiary care hospital with 60 beds in six ICUs. For this study, IPC measures included contact precautions (gloves, aprons, caps, and/or glasses), thorough disinfectant surface cleaning, hand hygiene, and strict antibiotic use control. The study spanned a duration of 18 months, commencing in December 2020 and concluding in May 2022, and was divided into two phases, each spanning a period of nine months. Phase 1, serving as control, encompassed 11,425 patient days, during which all patients admitted to ICUs underwent weekly ASC for MDR-AB, MDR-PA, and CRE. Phase 2, spanning 12,153 patient days, introduced ASC exclusively upon patient admission and discharge. ASC was obtained from the oropharynx or rectum of patients. Regardless of phase, all patients, including those with negative results, were kept under IPC measures until ICU discharge. For Phase 2, the decision was made to take swabs only upon the patients' admission and discharge to determine whether the case was imported or autochthonous. Patients were considered infected if they had a positive result in clinical material (blood, urine, or tracheal aspirate). They were kept in isolation and under IPC interventions until they were discharged from the ICU. No substantial alterations in patient health status, hospital infrastructure, or infection prevention techniques occurred at any of the sites during the study period.

The overall infection rate and the rate of infection for each pathogen in all ICUs were determined monthly. The BD Phoenix™ Automated Microbiology System (Becton, Dickinson and Company, USA) was used to identify the species of bacteria obtained from infections in the ICUs and to profile their antibiotic susceptibility. For laboratory purposes, the minimum inhibitory concentration of ertapenem, imipenem, and meropenem was determined for all MDR-AB, MDR-PA, and CRE isolates from clinical material, in accordance with the Clinical and Laboratory Standards Institute guidelines. Bacteria resistant to at least one antimicrobial drug from at least three antibiotic classes were considered multidrug resistant (MDR). The Poisson or negative binomial distribution (in case of over-dispersed data) models were used to compare the rates of bacterial infections during the study phases using R software. The results were expressed as an incidence rate ratio (IRR) with their corresponding 95% confidence interval (95%-CI) values.

## Results

We compared the infection rates of two patient cohorts under the same IPC measures (contact precautions, thorough disinfectant surface cleaning, hand hygiene, and strict antibiotic use control). The only variable was the frequency with which screening tests with swabs were performed. During the 18-month period of this study, there were 233 cases of MDR-GNB infections with 130 cases (55.7%) in Phase 1 and 103 (44.3%) in Phase 2. The overall infection rate per 1,000 patient-days was 11.38 (130/11,425) in Phase 1 and 8.47 (103/12,153) in Phase 2 (IRR: 1.34; CI 95% 1.04–1.74) (Table 1 (Tab. 1)). This is statistical evidence suggesting that Phase 1 had more infections per unit of time than Phase 2 for all MDR-GNB. Table I details the distribution of infections by species. Generally, there were no statistically significant differences between phases for individual species.

## Discussion

The increasing burden of MDR-GNB infections has underscored the pressing need for effective prevention and control strategies. Although the implementation of ASC programs remains a subject of debate, it may offer certain advantages, contingent on specific variables [5]. A notable benefit of an ASC program is its capacity to identify colonized patients, enabling their enrollment in contact precautions to prevent cross-transmission. This approach optimizes more appropriate empirical therapy, leading to a reduction in mortality and selection pressure [7], [8]. However, the high frequency of false-negative ASC samples may lead to underestimation of the true burden of MDR-GNB, resulting in ineffective containment and inadequate initial therapy [6]. Other limitations include low sensitivity for cultures from certain sites, labor intensity, high cost of selective plates, administrative difficulties, and low standardization of ASC policies [9], [10]. 

This study evaluated the effectiveness of performing ASC weekly versus only upon patient admission and discharge from the ICU. We observed that reducing surveillance through ASC did not result in an increase in infection rates. In fact, when considering all MDR-GNB evaluated, statistical evidence of a reduction in infection rates was observed in Phase 2. However, this may be due to the reduced ASC screening in Phase 2. In any case, conducting additional swab tests did not affect the control of bacteria in the ICU. Therefore, implementing several infection control measures in both phases was effective. This makes it possible to optimize hospital staff functions and decrease costs. Although there may be additional costs for contact precaution items, this protocol reduces costs associated with swabs, labor for collection, and laboratory expenses. Indeed, these results were similar to a study considering methicillin-resistant *Staphylococcus aureus* in a neonatal intensive care unit [11]. Further studies on ASC are necessary to determine the applicability of these results to all types of bacteria and hospital environments or only in specific cases.

Based on these results, we have changed the periodicity of ASC from weekly to admission and discharge of patients, and we have maintained contact precautions for all patients in our hospital. The Infection Control Committee and hospital management endorsed this modification to the ASC protocol in September 2021, and it has since been established as an institutional policy. As of June 2025, the stability of our infection and transmission rates indicates the efficacy of these measures.

### Limitations

This study has several limitations. First, it was conducted in a single tertiary hospital, which may limit the generalizability of the findings to other institutions or epidemiological settings. Second, only MDR-GNB (*A. baumannii, P. aeruginosa*, and CRE) were assessed, limiting applicability to other pathogens. Finally, the observation period of 18 months may not capture long-term epidemiological trends.

## Conclusions

Reducing ASC frequency from weekly to only upon ICU admission and discharge did not increase MDR-GNB infection rates. The implementation of comprehensive infection prevention and control measures proved sufficient for the management of bacterial infections. This approach has been demonstrated to reduce costs and optimize resource allocation. Further studies are needed to generalize these findings to other settings and pathogens.

## Notes

### Competing interests

The authors declare that they have no competing interests.

### Ethical approval 

This study analyzed anonymized, aggregated data generated from the hospital’s routine infection prevention and control program. No patient-identifiable information was collected, and no additional procedures were performed for research purposes. According to Brazilian National Research Ethics Guidelines (CNS Resolution 466/12), such studies are exempt from ethics committee review.

### Funding 

This study was financed by FAPERJ - Fundação Carlos Chagas Filho de Amparo à Pesquisa do Estado do Rio de Janeiro, Processo SEI-260003/019688/2022.

### Acknowledgments

We thank Fabiano R. Oliveira for administrative support.

### Authors’ ORCIDs

- Lima L: https://orcid.org/0000-0003-3921-560X
- Alves E: https://orcid.org/0009-0006-2636-5835
- SantosB: https://orcid.org/0000-0001-6841-5104
- Mascarenhas J: https://orcid.org/0009-0007-0681-7758
- Rizzo C: https://orcid.org/0009-0000-3662-784X
- Morgado S: https://orcid.org/0000-0003-4877-7639
- Mascarenhas L: https://orcid.org/0000-0002-0500-1219

## Figures and Tables

**Table 1:**
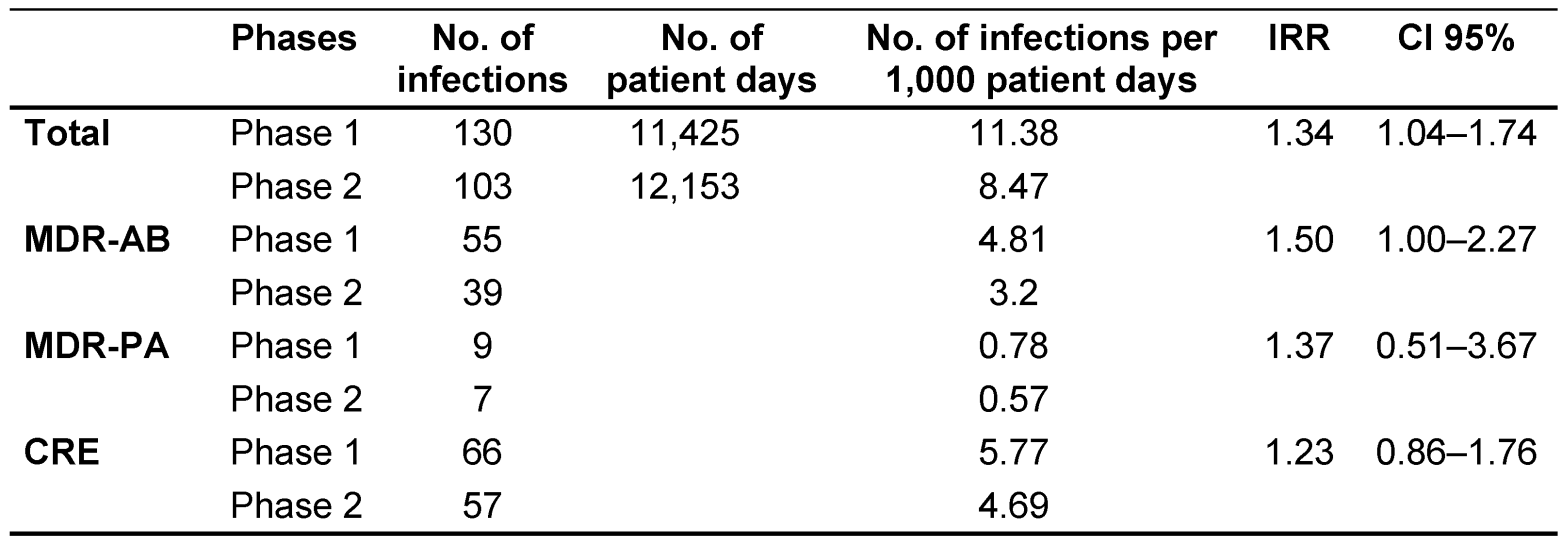
Rates of MDR-GNB infections
